# Regional variation in post‐operative mortality in New Zealand

**DOI:** 10.1111/ans.17510

**Published:** 2022-04-20

**Authors:** Jason K. Gurney, Melissa McLeod, James Stanley, Bridget Robson, Douglas Campbell, Elizabeth Dennett, Dick Ongley, Juliet Rumball‐Smith, Diana Sarfati, Jonathan Koea

**Affiliations:** ^1^ Department of Public Health University of Otago Wellington New Zealand; ^2^ Auckland District Health Board Auckland New Zealand; ^3^ Capital and Coast District Health Board Wellington New Zealand; ^4^ Canterbury District Health Board Christchurch New Zealand; ^5^ Ministry of Health Wellington New Zealand; ^6^ Te Aho o Te Kahu – Cancer Control Agency Wellington New Zealand; ^7^ Waitemata District Health Board Auckland New Zealand

**Keywords:** disparity, inequity, perioperative mortality, post‐operative mortality, regional variation

## Abstract

**Background:**

There is a growing body of evidence that access to best practice perioperative care varies within our population. In this study, we use national‐level data to begin to address gaps in our understanding of regional variation in post‐operative outcomes within New Zealand.

**Methods:**

Using National Collections data, we examined all inpatient procedures in New Zealand public hospitals between 2005 and 2017 (859 171 acute, 2 276 986 elective/waiting list), and identified deaths within 30 days. We calculated crude and adjusted rates per 100 procedures for the 20 district health boards (DHBs), both for the total population and stratified by ethnicity (Māori/European). Odds ratios comparing the risk of post‐operative mortality between Māori and European patients were calculated using crude and adjusted Poisson regression models.

**Results:**

We observed regional variations in post‐operative mortality outcomes. Māori, compared to European, patients experienced higher post‐operative mortality rates in several DHBs, with a trend to higher mortality in almost all DHBs. Regional variation in patterns of age, procedure, deprivation and comorbidity (in particular) largely drives regional variation in post‐operative mortality, although variation persists in some regions even after adjusting for these factors. Inequitable outcomes for Māori also persist in several regions despite adjustment for multiple factors, particularly in the elective setting.

**Conclusions:**

The persistence of variation and ethnic disparities in spite of adjustment for confounding and mediating factors suggests that multiple regions require additional resource and support to improve outcomes. Efforts to reduce variation and improve outcomes for patients will require both central planning and monitoring, as well as region‐specific intervention.

## Graphical abstract

In this study, we used national‐level data to begin to address gaps in our understanding of regional variation in post‐operative outcomes within New Zealand. After adjusting for confounding, we found regional variation in post‐operative mortality and in equity of outcomes between Māori and European patients. Regional variation in patterns of age, deprivation and comorbidity (in particular) helps to drive regional variation in post‐operative mortality, although variation persists in some regions even after adjusting for these factors.

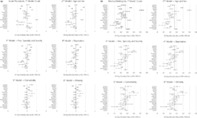

## Introduction

At the level of individual care, death shortly after surgery may occur despite timely, high‐quality care being provided. At the population level, patterns of deaths shortly after surgery may be used to indicate potential inadequacies in access to (and delivery of) high‐quality perioperative care.^1^ At a systems level, these inadequacies may relate to (i) the resourcing and availability of ‘prehabilitation’ services aimed at optimizing a patient's condition prior to surgery; (ii) the systematic and consistent use of robust clinical checklists (and broader pathways); (iii) resourcing of high‐quality post‐operative management, including management of comorbidities; and (iv) the selection of the operative (e.g. high‐volume versus low‐volume hospital) and post‐operative environment (e.g. inside versus outside the hospital).^1^, ^2^, ^3^

Within the context of publicly funded surgery in New Zealand, there is a growing body of evidence that access to best practice perioperative care varies – sometimes strikingly – within our population. Clear disparities in post‐operative outcomes have been identified for Māori patients relative to European patients,^4^, ^5^, ^6^, ^7^, ^8^ and there is also evidence that New Zealanders living in deprivation are at an increased risk of post‐operative mortality.^4^

However, few studies have examined geographical variation in post‐operative outcomes within New Zealand. When recently examining variation in 30‐day mortality following hip fracture repair between regions, New Zealand's Perioperative Mortality Review Committee (POMRC) found that once rates were adjusted for age, gender, ethnicity, deprivation and comorbidity, there was no substantive difference between district health boards (DHBs).^9^ However, Lao *et al*.^10^ observed substantial differences in the length of stay following hip and knee replacement surgeries across New Zealand, with the authors noting that this may be driven by regional variation in access to specialist surgeons. Outside of orthopaedic surgery, there is evidence that in some contexts there may be variation in the quality of surgical care depending on where you live in New Zealand. For example, Signal *et al*.^11^ found that Māori patients with stomach cancer were less likely to have access to specialist surgeons for their gastric resection, with this disparity likely driven by differences where Māori and non‐Māori patients are accessing care. However, the extent of any such regional variation remains largely unknown.

In the current study, we used national‐level data to begin to address gaps in our understanding of regional variation in post‐operative outcomes within New Zealand. We aimed to address the following questions: (i) to what extent does the rate of 30‐day post‐operative mortality differ between region of treatment in New Zealand?, (ii) to what extent can any variation be explained by differences in procedure‐ and patient‐related factors between regions? and finally (3) given what we understand about ethnic inequities in post‐operative mortality within New Zealand, do Māori and European patients experience similar regional variation in 30‐day post‐operative mortality?

## Methods

Our study cohort included all patients who underwent an inpatient procedure in a New Zealand public hospital between 1 January 2005 and 31 December 2017, as recorded on the National Minimum Dataset (NMDS).^12^ We restricted our analysis to New Zealand residents to ensure follow‐up for post‐operative death, excluded patients with an ASA (American Society of Anesthesiologists) score of 6 and excluded patients whose procedure was not publicly funded (i.e. not funded by a DHB).

### Variables

DHB of treatment was defined as the DHB in which the procedure took place, and was determined using the hospital facility code within the NMDS. Procedure specialty was determined by mapping procedures to the Australasian College of Health Informatics (ACHI) procedure code ‘block’, which is organized by anatomical specialty.^13^ Procedure risk was established using a modified version^14^ of the Johns Hopkins Surgical Risk Classification System,^15^ which classifies surgical risk into five categories according to factors including the invasive nature of the procedure and potential for blood loss.^14^ All analyses were stratified by admission type, which was categorized as either acute or elective/waiting list based on NMDS data.

Patient comorbidity was measured in two ways: using the ASA physical status score (to measure acute morbidity at the time of procedure) and the M3 index of multimorbidity (to measure long‐term condition morbidity in the build‐up to the procedure).^16^ For the M3 index, NMDS data from 5 years prior to admission were coded for the presence of any of the 61 M3 conditions using International Classification of Diseases (ICD‐10‐AM) codes, which were then weighted and summed to arrive at the M3 score.^16^ M3 scores were included as a splined variable within Poisson models with knots at the 0th, 90th and 95th percentiles.^17^ ASA score was determined from ICD anaesthesia codes at the time of the procedure, and categorized as either 1–2 (healthy or mild/moderate disease), 3 (severe but stable disease), 4–5 (severe disease with immediate threat to life) or unknown.^5^ Date of death was defined using the National Health Index (NHI) data set.^18^ Ethnicity data were from the NHI records and categorized in the prioritized order of Māori, Pacific, Asian, European or Middle Eastern/Latin American/African/Other (hereafter MELAA/other) to generate mutually exclusive groups.^19^ For the purposes of this analysis, and given known disparities in post‐operative mortality between Māori and European patients,^4^, ^5^ we focussed on Māori and European ethnic groups.

### Statistical analysis

Crude descriptive analysis was used to determine the number and rate (per 100 procedures) of death within 30 days of any procedure, stratified by DHB. When examining ethnicity‐specific rates of post‐operative mortality, we determined age‐standardized rates (per 100 procedures) using direct standardization methods,^20^ with the total Māori surgical population during 2005–2017 (all procedures; 528 517) as the standard population. We chose this standard population for two reasons: (i) the underlying age structure of this population largely reflects that of the Māori patients in the current study and (ii) we believe using an Indigenous standard population is a best practice approach when comparing Māori to other ethnic groups.^21^, ^22^

To examine the impact of the potential drivers of variation in rates of post‐operative mortality between regions, we calculated both crude and adjusted rates stratified by DHB using Poisson regression. Rates were calculated as the number of deaths within the 30‐day post‐operative period (including the date of the procedure), as a function of the total number of procedures that were performed over the follow‐up period as the denominator. Covariates (or ‘explanatory variables’) were added in a step‐by‐step manner to the Poisson models – starting with the crude (unadjusted) model that compared rates by DHB (first model), then iteratively adding age and sex as classic confounders (second model), then variables relating to the type of procedure being undertaken (procedure specialty and procedure risk, third model), a variable representing socioeconomic deprivation (NZDep quintile, fourth model), then two variables representing chronic and acute comorbidity (M3 score and ASA category respectively, fifth model) and finally ethnicity (sixth model). We adjusted for ethnicity last, in order to ensure that the impact of comorbidity and deprivation was already accounted for within the model, given the differential burden of these factors between ethnicities.^23^, ^24^ The corresponding per‐DHB rates from each adjusted model were calculated using marginal standardization (i.e. standardizing the rate as though each DHB had the national‐level covariate profile) using Stata's margins command.^25^

In addition to completing the above analysis for the total cohort, we also stratified our results by ethnicity, with a focus on Māori and European ethnic groups. Using crude and adjusted rates of post‐operative death for Māori and European patients, we calculated rate ratios between ethnic groups, stratified by DHB. We ran the same iterative models as for the total population (with the exception of ethnicity). We did not compare ethnic groups in those DHBs where fewer than 10 Māori patients died over the follow‐up period, in an effort to avoid over‐interpretation of imprecise data.

Data management and analysis was completed in SAS v9.4 (SAS Institute, USA), Stata v16 (StataCorp LLC, USA) and Microsoft Excel 2016 (Microsoft Corp., USA). Ethical approval for this study was sought and obtained from the University of Otago Human Ethics Committee (Health), approval # HD18/085.

## Results

The number of procedures and deaths are presented in Table 1, both for the total population and stratified by DHB. A total of 3 136 157 procedures that occurred between 2005 and 2017 were included in our analysis (859 171 acute procedures; 2 276 986 elective/waiting list procedures). Over this period, a total of 23 272 deaths were recorded within 30 days of a procedure (17 175 within 30 days of an acute procedure, crude rate 2.00/100 procedures; 6097 within 30 days of an elective/waiting list procedure, crude rate 0.27/100 procedures). Procedures and deaths were distributed in line with the national population structure and locations of tertiary hospitals, with the greatest volume of procedures and post‐operative deaths occurring in Auckland (523 434 procedures, 4970 deaths), Canterbury (402 992 procedures, 2545 deaths), Waikato (280 877 procedures, 3268 deaths), Counties‐Manukau (277 996 procedures, 1553 deaths) and Capital and Coast (229 941 procedures, 2159 deaths) DHBs.

**Table 1:** Number of procedures and deaths and the crude rate (*n*/100 procedures) of 30‐day mortality following any publicly funded inpatient surgical procedure in New Zealand performed between 2005 and 2017 by DHB

	Acute procedures			Elective/waiting list procedures		
	Procedures	30‐day mortality		Procedures	30‐day mortality	
	*n*	*n*	*n*/100	*n*	*n*	*n*/100
National total	859 171	17 175	2.00	2 276 986	6097	0.27
DHB						
Northland	28 163	592	2.10	72 031	172	0.24
Waitemata	62 298	902	1.45	156 285	258	0.17
Auckland	144 047	3341	2.32	379 387	1329	0.35
Counties‐Manukau	94 628	1207	1.28	183 368	346	0.19
Waikato	91 380	2292	2.51	189 497	976	0.52
Lakes	20 592	347	1.69	43 935	124	0.28
Bay of Plenty	38 073	695	1.83	97 937	200	0.20
Tairawhiti	8599	163	1.90	21 316	45	0.21
Hawke's Bay	33 624	643	1.91	84 243	200	0.24
Taranaki	19 049	401	2.11	63 927	151	0.24
MidCentral	31 000	548	1.77	76 890	194	0.25
Whanganui	12 267	225	1.83	38 176	65	0.17
Capital and Coast	52 839	1585	3.00	177 102	574	0.32
Hutt Valley	35 212	331	0.94	74 433	104	0.14
Wairarapa	5063	73	1.44	17 077	23	0.13
Nelson/Marlborough	17 358	385	2.22	84 609	132	0.16
West Coast	3537	44	1.24	13 277	10	0.08
Canterbury	94 496	1901	2.01	308 496	644	0.21
South Canterbury	8386	184	2.19	33 422	55	0.16
Southern	58 560	1316	2.25	161 578	495	0.31

DHB, district health board.

**Fig. 1:**
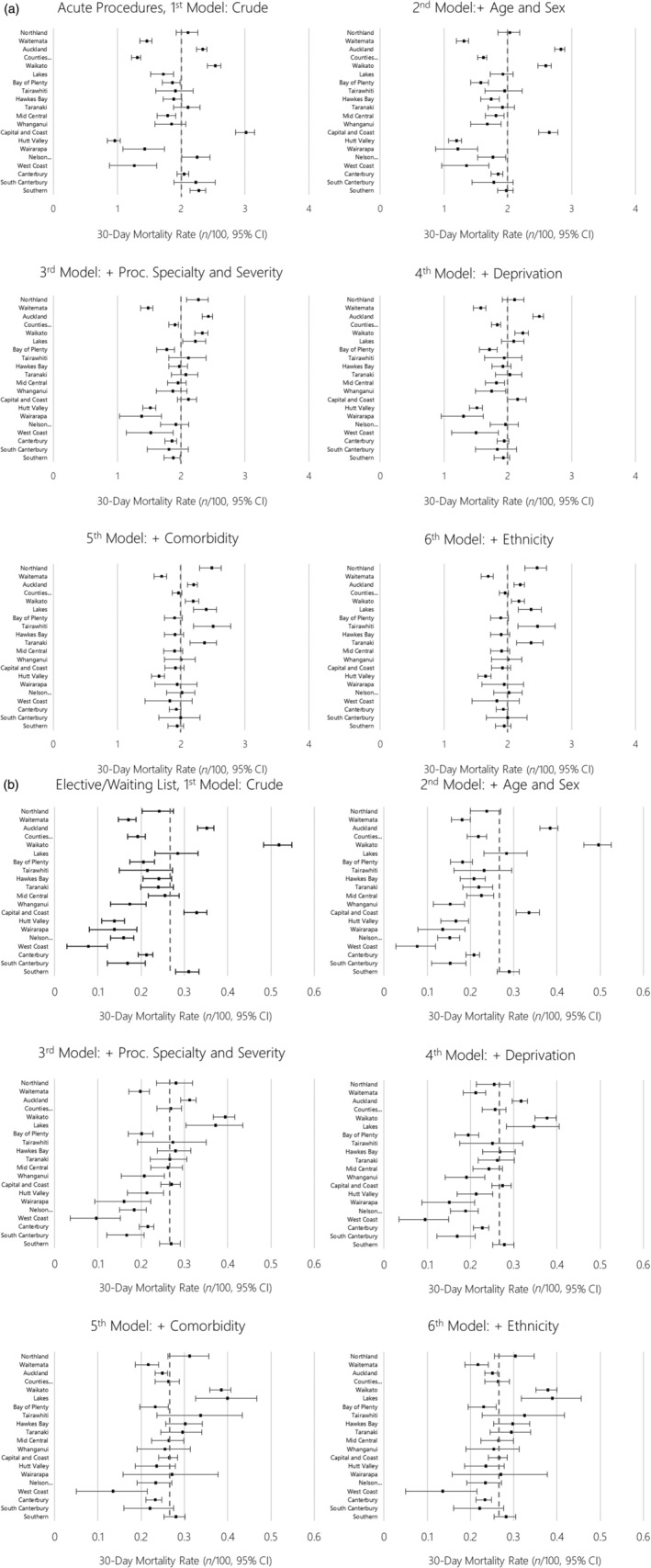
Rate of 30‐day mortality following (a) acute and (b) elective/waiting list procedures, by district health boards, with iterative adjustment for modelled variables. The dashed grey line is the crude national rate.

Figures 1a (acute) and 1b (elective/waiting list) compare the rate of post‐operative mortality observed for each DHB with the national rate, iteratively adjusted for covariates, with these data also presented in Table S1. We found that adjusting for age and sex had a greater impact on the rates of post‐operative mortality following acute admission than for elective/waiting list procedures. We also noted that adjusting for age and sex had a variable impact on the observed rates between DHBs. Adjustment for procedure specialty and severity strongly reduced the rate of post‐operative mortality for some treatment hubs (e.g. Auckland DHB), and also tended to reduce variation between DHBs around the national average rate for both acute and elective/waiting list procedures. Adjustment for deprivation tended to further reduce this variation for acute procedures, but less so for elective/waiting list procedures. Adjustment for comorbidity tended to increase the observed rate of post‐operative mortality in several small centres, particularly for acute procedures (e.g. Northland, Lakes, Tairawhiti and Taranaki), while simultaneously tending to further reduce variation around the national average rate. After adjusting for all these factors, further adjustment for ethnicity appeared to have little impact on the rates of post‐operative mortality across the country.

Ethnicity‐stratified data are presented in Table 2, both for the total population and stratified by DHB. For Māori, a total of 3150 deaths were recorded over this time period (2267 within 30 days of an acute procedure, age‐standardized rate 1.59/100 procedures; 883 within 30 days of an elective/waiting list procedure, crude rate 0.25/100 procedures). For Europeans, a total of 18 060 deaths were recorded over this time period (13 349 within 30 days of an acute procedure, age‐standardized rate 0.98/100 procedures; 4711 within 30 days of an elective/waiting list procedure, crude rate 0.13/100 procedures).

**Table 2:** Number of deaths, crude and age‐standardized rates (per 100 procedures) of 30‐day mortality following (a) acute and (b) elective/waiting list procedures in New Zealand, by DHB, separately for Māori and European patients

(a) Acute procedures								
	Māori – acute				European – acute			
	Procedures	Deaths	Crude rate	Age‐standardized rate	Procedures	Deaths	Crude rate	Age‐standardized rate
	*n*	*n*	*n*/100	*n*/100	*n*	*n*	*n*/100	*n*/100
National total	161 337	2267	1.41	1.59	571 496	13 349	2.34	0.98
DHB								
Northland	10 465	132	1.26	1.29	16 950	455	2.68	0.82
Waitemata	6621	46	0.69	0.75	45 124	768	1.70	0.49
Auckland	20 123	555	2.76	3	78 608	2043	2.60	1.62
Counties‐Manukau	22 686	206	0.91	1.27	39 861	726	1.82	0.71
Waikato	25 376	519	2.05	2.09	59 971	1692	2.82	1.17
Lakes	8213	84	1.02	1.13	11 455	258	2.25	0.7
Bay of Plenty	10 442	77	0.74	0.81	26 380	608	2.30	0.61
Tairawhiti	4414	45	1.02	0.98	3894	117	3.00	0.85
Hawke's Bay	9718	111	1.14	1.31	21 947	510	2.32	0.64
Taranaki	3414	32	0.94	1.03	14 798	360	2.43	0.74
Mid Central	5371	44	0.82	1	24 004	496	2.07	0.75
Whanganui	3180	28	0.88	1.05	8733	196	2.24	0.59
Capital and Coast	7712	182	2.36	2.25	37 279	1240	3.33	1.64
Hutt Valley	7889	23	0.29	0.56	22 051	278	1.26	0.49
Wairarapa	902	4	0.44	—	3998	69	1.73	0.67
Nelson/Marlborough	1574	17	1.08	1.26	15 272	363	2.38	0.67
West Coast	377	6	1.59	—	3062	38	1.24	0.44
Canterbury	7408	120	1.62	2.09	79 363	1694	2.13	0.96
South Canterbury	537	5	0.93	—	7633	178	2.33	0.72
Southern	4915	31	0.63	0.73	51 113	1260	2.47	0.98

(b) Elective/waiting list procedures								
	Māori – elective/waiting list				European – elective/waiting list			
	Procedures	Deaths	Crude rate	Age‐standardized rate	Procedures	Deaths	Crude rate	Age‐standardized rate
	*n*	*n*	*n*/100	*n*/100	*n*	*n*	*n*/100	*n*/100
National total	340 053	883	0.26	0.25	1 616 928	4711	0.29	0.13
DHB								
Northland	24 600	53	0.22	0.19	45 429	116	0.26	0.08
Waitemata	14 005	15	0.11	0.11	111 969	230	0.21	0.08
Auckland	46 804	209	0.45	0.45	217 188	844	0.39	0.21
Counties‐Manukau	32 453	86	0.26	0.26	84 556	193	0.23	0.1
Waikato	41 412	188	0.45	0.39	134 893	734	0.54	0.22
Lakes	15 151	31	0.20	0.17	26 596	92	0.35	0.1
Bay of Plenty	22 204	31	0.14	0.13	71 692	169	0.24	0.05
Tairawhiti	9622	14	0.15	0.12	11 093	30	0.27	0.08
Hawke's Bay	19 728	38	0.19	0.15	59 580	157	0.26	0.08
Taranaki	9466	14	0.15	0.15	52 250	134	0.26	0.09
Mid Central	10 428	26	0.25	0.22	62 404	167	0.27	0.1
Whanganui	8302	15	0.18	0.15	28 803	50	0.17	0.08
Capital and Coast	23 343	85	0.36	0.35	123 923	442	0.36	0.18
Hutt Valley	11 869	10	0.08	0.04	52 375	89	0.17	0.05
Wairarapa	2561	1	0.04	—	13 911	22	0.16	0.05
Nelson/Marlborough	7319	4	0.05	—	74 320	128	0.17	0.06
West Coast	1109	0	0.00	—	11 867	10	0.08	0.07
Canterbury	25 049	43	0.17	0.2	261 769	581	0.22	0.11
South Canterbury	1814	1	0.06	—	30 811	53	0.17	0.05
Southern	12 814	19	0.15	0.15	141 499	470	0.33	0.14

Rates were not calculated for DHBs where the number of deaths is <10.

DHB, district health board.

**Fig. 2:**
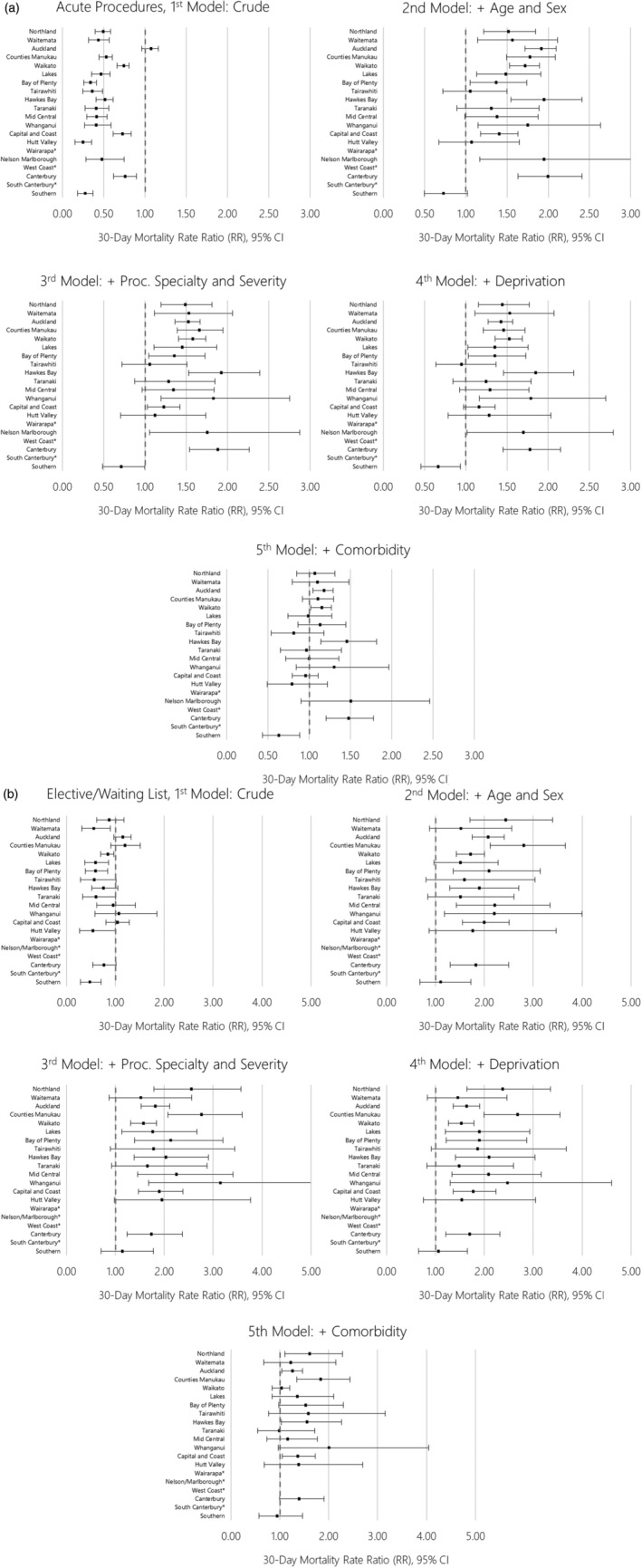
Māori versus European 30‐day mortality rate ratios following (a) acute and (b) elective/waiting list procedures, by district health boards, with iterative adjustment for modelled variables. *Data not shown due to sparse data.

The relative risks of post‐operative mortality between Māori and European patients are shown in Figures 2a (acute) and 2b (elective/waiting list), with these data also presented in Table S2. Disparities in post‐operative mortality broadly ranged (in the age‐ and sex‐adjusted model) from 30% to 100% increased risk following acute procedures, to 50–150% increased risk following elective/waiting list procedures. With the exception of Auckland DHB, adjustment for procedure specialty or severity had minimal impact on disparities between Māori and European patients within DHBs for both acute and elective/waiting list procedures. Adjustment for deprivation also had minimal impact. Adjustment for patient comorbidity substantially attenuated the observed disparities between Māori and European patients for most DHBs, while simultaneously reducing variation in this disparity across DHBs. However, disparities in post‐operative mortality between Māori and European patients remained within several DHBs after adjusting for all included covariates. For example, post‐operative mortality following acute procedures remained 40–50% higher among Māori patients in Hawke's Bay, Nelson‐Marlborough and Canterbury DHBs after adjustment for all included covariates, and for elective/waiting list procedures was 30–100% higher in Northland, Counties‐Manukau, Bay of Plenty, Whanganui and Capital and Coast DHBs.

## Discussion

To date, there has been limited research regarding regional variation in post‐operative outcomes in New Zealand. In this national study of all publicly funded inpatient procedures over a 13‐year period, we found rates of mortality following acute (2/100 procedures) and elective/waiting list (0.27/100 procedures) are in line with those observed in other regions including the USA.^26^ However, we observed substantial variation between regions: crude rates of post‐operative mortality following acute procedures ranged from 0.94/100 to 3.00/100 procedures (national rate 2.00/100), and for elective/waiting list procedures from 0.08/100 to 0.52/100 (national rate 0.27/100). The majority of this regional variation could be explained by our key explanatory factors: adjusting for differential patterns of demographic factors including age and deprivation, potential differences in the types of procedures performed between regions, and differential patterns of patient comorbidity, tended to reduce the observed variation for rates for any given region around the national rate. However, even after adjusting for differences in these procedure‐ and patient‐level factors, some DHBs had a higher rate of post‐operative mortality compared to the national average. These DHBs tended to be smaller (e.g. Tairawhiti) and/or to serve large populations with a high proportion of Māori residents (e.g. Northland, Waikato and Lakes). This finding might suggest that insufficient resources are being directed towards regions with high proportions of Māori patients; however, further nuanced investigation of the reasons for higher residual mortality in these DHBs is required. We also note that further Māori health expertise, perhaps situated within the new Māori Health Authority, will be a necessary component of solutions aimed at reducing post‐operative mortality within regions that have a large Māori population.

We found that some of the initially observed higher rates of post‐operative mortality in regions such as Auckland and Capital and Coast DHBs were explained by differences in both the type and risk of the procedure. This suggests that most of the excess risk identified in the age‐/sex‐adjusted models was likely due to these DHBs performing more higher‐risk procedures; for example, coronary artery bypass graft procedures are only performed within the five main centres (data not shown).

Adjusting for deprivation had limited impact on the rates of 30‐day death within the majority of DHBs, and had little or no impact on disparities between Māori and European patients with the exception of Northland and Lakes DHBs. This highlights that it is entirely feasible that some covariates will modify the risk of post‐operative mortality in some regions, but not in others.

Even after adjusting for the impact of differential patterning of age, sex, procedure type/severity and deprivation, comorbidity had possibly the strongest impact on both rates of 30‐day death in the total cohort (Fig. 1) and disparities between Māori and Europeans (see below). This strong impact is consistent with evidence that comorbidity is an important driver of post‐operative mortality in New Zealand,^4^, ^8^ and therefore the perioperative management of patients with comorbidity (including prehabilitation) is a crucial determinant of post‐operative outcomes. It also highlights the importance of equitable access to primary and secondary prevention through strong public health policy and primary care as a means of improving perioperative outcomes at a population level.

Once adjusted for the confounding impact of the younger age structure of the Māori population, we observed substantial disparities in post‐operative mortality between Māori and European patients across nearly all DHBs (Fig. 2). While each included covariate did explain some of this disparity – and the extent of that explanation varied by DHB – the strongest observed driver of disparities in post‐operative mortality within DHBs was comorbidity. This observation further emphasizes the role of comorbidity as an independent driver of post‐operative mortality, as well as a key driver of inequities in perioperative outcomes between Māori and European patients. It is also important to note that the importance of factors such as comorbidity – and, by connection, procedure type and risk – is related to differential access to the social determinants of health for Māori patients. As such, these factors might be conceptualized in this context as potential examples of the role of institutionalized racism as a driver of differential perioperative outcomes for Māori in New Zealand – a bias whereby the systems that underpin society work better for some groups than they do for others.^27^

It is important to note that an absence of disparities (or of variation between DHBs in the rates of 30‐day death) following adjustment for covariates such as comorbidity does not translate to an absence of room for improvement within a given region; merely that the included covariates were, in many cases, able to explain the majority of this variation. Some of the key actions required to reduce the burden of comorbidity or increase the equitable distribution of care throughout the country need to occur centrally – including changes to how the health system is structured following the Health and Disability System Review,^28^ and the potential for the new Māori Health Authority to monitor and intervene to address systemic racism and inequities across the health system, which could include additional resourcing of Māori health providers at a regional level. In addition to these centralized activities, each region must consider its own unique challenges – those factors that may feasibly increase the risk of post‐operative mortality, such as a high population burden of comorbidity – implement solutions to these challenges and then monitor perioperative outcomes (possibly with the assistance of central government) to see what is and is not working. There is also of value in the ongoing measurement of variation between DHBs in perioperative outcomes, as a means of quality improvement (as is currently being rolled‐out in the context of cancer care).^29^, ^30^, ^31^ This monitoring enables us to identify those regions who are performing the best, so that they can provide examples of good practice and support to those regions with poorer performance. The POMRC is well positioned to continue this vital monitoring service, if it is resourced to do so effectively.

Upcoming changes to the structure of New Zealand's health system following the Health and Disability System Review^28^ will invariably alter future choices for how we need to conceptualize and examine regional variation in health service delivery. Part of this examination will require us to consider our tolerance for regional variation that cannot be explained by factors that are relatively fixed – such as age – and exactly what that tolerance level should be. However, we note that the findings outlined here will remain relevant regardless of future structure: (i) that (unadjusted) regional variation in post‐operative mortality is inevitable given higher‐risk procedures are only performed in some centres; (ii) that regional variation in patterns of age, deprivation and particularly comorbidity also help to drive regional variation in post‐operative mortality; (iii) that regional variation in post‐operative mortality persists in some regions even after adjusting for all of these explanatory factors; (iv) that Māori have higher rates of post‐operative mortality than Europeans in nearly every region across the country, and that this disparity is most strongly driven by the disproportionate burden of comorbidity shouldered by Māori patients; and (v) that this ethnic disparity persists in several regions despite adjustment for possible confounding and mediating factors. It is therefore imperative that the post‐reform health system structure in New Zealand, which will include a Māori Health Authority, seeks to maximize equity while recognizing that regional differences in need might require differing levels of resource investment, including additional investment in Māori health providers. It is likely that such solutions will need to take into account of factors that sit outside of health but which may also vary by region, such as the quality of education, housing and other key systemic challenges that may be differentially faced by regions with large Māori populations.

### Study strengths and limitations

This study provides a national‐level overview of post‐operative mortality following publicly funded inpatient procedures within New Zealand. We used national routinely collected health records for all procedures performed in New Zealand over a 13‐year period, which enhanced the validity and generalizability of our findings. We note that this study relies on the accuracy of routinely collected health records, as reported by DHBs to the Ministry of Health. We also note that as this is a study of regional variation in post‐operative outcomes following a publicly funded procedure, and because of limitations in access to consistent and comprehensive privately funded surgical data at a national level, this study does not consider regional variation in outcomes following a privately funded procedure. We note that while we have included multiple explanatory factors within our analysis, there are likely to be other factors that could contribute to regional variation (as well as variation in ethnic disparity) that we cannot examine with the available data (e.g. regional variation in case selection, waiting list burdens and so on). Finally, we note that the current study grouped all procedures together, and that the extent of regional variation in post‐operative outcomes may differ between procedure types; further research is required to examine regional variation by surgical specialty.

## Conclusions

We found that there is both regional variation in post‐operative mortality after adjusting for multiple confounders (including age, sex, procedure type and complexity), as well as regional variation in equity of outcomes between Māori and European patients. The persistence of variation and ethnic disparities in spite of adjustment for confounding and mediating factors (including procedure type and comorbidity) suggests that there is room for improvement in multiple regions. Efforts to reduce variation and improve outcomes for patients will require both central planning and monitoring, as well as region‐specific reflection and intervention.

## Author contributions

**Jason K. Gurney:** Conceptualization; data curation; formal analysis; funding acquisition; investigation; methodology; project administration; supervision; writing – original draft; writing – review and editing. **Melissa McLeod:** Conceptualization; investigation; writing – review and editing. **James Stanley:** Formal analysis; investigation; methodology; writing – review and editing. **Bridget Robson:** Investigation; writing – review and editing. **Douglas Campbell:** Investigation; writing – review and editing. **Elizabeth Dennett:** Investigation; writing – review and editing. **Dick Ongley:** Investigation; writing – review and editing. **Juliet Rumball‐Smith:** Investigation; writing – review and editing. **Diana Sarfati:** Investigation; writing – review and editing. **Jonathan Koea:** Conceptualization; investigation; writing – review and editing.

## Conflict of interest

None declared.

## Supporting information


**Table S1.** Crude and adjusted rate of 30‐day mortality (*n*/100) following (a) acute and (b) elective/waiting list procedures, iteratively adjusted for potential drivers of variation between district health boards (i.e. tabulation of data presented in Fig. 1).
**Table S2.** Māori versus European 30‐day mortality rate ratios following (a) acute and (b) elective/waiting list procedures, by district health board (DHB), with iterative adjustment for modelled variables (i.e. tabulation of data presented in Fig. 2). Data are not presented for those DHBs where fewer than 10 Māori deaths occurred over the study period.
