# Supplementary material for: Regional variation in post‐operative mortality in New Zealand
Source: ANZ J Surg. 2022 Apr 20;92(5):1015–25. doi: 10.1111/ans.17510 (PMC9321085; doi:10.1111/ans.17510)
Supplement: Supplementary file 1 — Table S1. Crude and adjusted rate of 30‐day mortality (n/100) following (a) acute and (b) elective/waiting list procedures, iteratively adjusted for potential drivers of variation between district health boards (i.e. tabulation of data presented in Fig. 1). Table S2. Māori versus European 30‐day mortality rate ratios following (a) acute and (b) elective/waiting list procedures, by district health board (DHB), with iterative adjustment for modelled variables (i.e. tabulation of data presented in Fig. 2). Data are not presented for those DHBs where fewer than 10 Māori deaths occurred over the study period. [file ANS-92-1015-s001.docx]

**Supplementary Material 1:** Crude and adjusted rate of 30-day mortality (n/100) following a) acute and b) elective/waiting list procedures, iteratively adjusted for potential drivers of variation between DHBs (i.e. tabulation of data presented in Figure 1a and 1b).

1. **Acute Procedures:**

|  | ***Crude*** | ***+ Age, Sex*** | ***+ Specialty, Risk*** | ***+ Deprivation*** | ***+ Comorbidity*** | ***+ Ethnicity*** |
| --- | --- | --- | --- | --- | --- | --- |
|  | n/100 (95% CI) | n/100 (95% CI) | n/100 (95% CI) | n/100 (95% CI) | n/100 (95% CI) | n/100 (95% CI) |
| **National Total** | 2.01 (1.98-2.04) | - | - | - | - | - |
|  |  |  |  |  |  |  |
| **District Health Board** | |  |  |  |  |  |
| *Northland* | 2.09 (1.92-2.26) | 2.01 (1.85-2.18) | 2.25 (2.07-2.44) | 2.09 (1.91-2.26) | 2.46 (2.26-2.66) | 2.44 (2.24-2.64) |
| *Waitemata* | 1.44 (1.35-1.54) | 1.29 (1.21-1.38) | 1.46 (1.36-1.56) | 1.56 (1.45-1.66) | 1.67 (1.56-1.79) | 1.67 (1.56-1.79) |
| *Auckland* | 2.32 (2.24-2.4) | 2.81 (2.72-2.91) | 2.41 (2.32-2.49) | 2.48 (2.39-2.57) | 2.18 (2.1-2.26) | 2.18 (2.1-2.26) |
| *Counties-Manukau* | 1.29 (1.22-1.37) | 1.6 (1.51-1.69) | 1.88 (1.78-1.99) | 1.82 (1.71-1.92) | 1.94 (1.82-2.05) | 1.94 (1.82-2.05) |
| *Waikato* | 2.51 (2.41-2.62) | 2.57 (2.47-2.68) | 2.31 (2.22-2.41) | 2.22 (2.12-2.31) | 2.17 (2.08-2.26) | 2.16 (2.07-2.25) |
| *Lakes* | 1.7 (1.52-1.88) | 1.9 (1.7-2.1) | 2.21 (1.97-2.44) | 2.08 (1.86-2.3) | 2.37 (2.12-2.62) | 2.35 (2.1-2.6) |
| *Bay of Plenty* | 1.85 (1.71-1.99) | 1.56 (1.44-1.68) | 1.75 (1.62-1.89) | 1.69 (1.56-1.82) | 1.88 (1.73-2.02) | 1.87 (1.73-2.01) |
| *Tairawhiti* | 1.89 (1.6-2.19) | 1.93 (1.64-2.23) | 2.1 (1.77-2.42) | 1.93 (1.63-2.23) | 2.48 (2.1-2.87) | 2.45 (2.07-2.83) |
| *Hawke's Bay* | 1.86 (1.72-2.01) | 1.72 (1.58-1.86) | 1.95 (1.8-2.11) | 1.9 (1.75-2.05) | 1.89 (1.74-2.04) | 1.88 (1.73-2.03) |
| *Taranaki* | 2.09 (1.88-2.29) | 1.9 (1.71-2.09) | 2.05 (1.85-2.25) | 2.01 (1.81-2.21) | 2.35 (2.11-2.58) | 2.35 (2.11-2.58) |
| *MidCentral* | 1.77 (1.62-1.92) | 1.8 (1.65-1.95) | 1.93 (1.77-2.09) | 1.8 (1.65-1.95) | 1.87 (1.72-2.03) | 1.88 (1.72-2.04) |
| *Whanganui* | 1.83 (1.59-2.07) | 1.66 (1.44-1.87) | 1.85 (1.61-2.09) | 1.73 (1.5-1.96) | 1.98 (1.72-2.24) | 1.98 (1.72-2.24) |
| *Capital and Coast* | 3 (2.85-3.15) | 2.63 (2.5-2.76) | 2.09 (1.99-2.2) | 2.14 (2.03-2.25) | 1.89 (1.8-1.99) | 1.9 (1.8-1.99) |
| *Hutt Valley* | 0.94 (0.84-1.05) | 1.18 (1.05-1.3) | 1.5 (1.34-1.66) | 1.5 (1.34-1.66) | 1.63 (1.46-1.81) | 1.63 (1.46-1.81) |
| *Wairarapa* | 1.41 (1.08-1.74) | 1.2 (0.92-1.48) | 1.36 (1.04-1.68) | 1.29 (0.99-1.59) | 1.92 (1.47-2.36) | 1.92 (1.47-2.37) |
| *Nelson/Marlborough* | 2.23 (2.01-2.45) | 1.75 (1.57-1.92) | 1.9 (1.71-2.09) | 1.94 (1.75-2.14) | 1.99 (1.79-2.2) | 2 (1.8-2.2) |
| *West Coast* | 1.25 (0.88-1.62) | 1.34 (0.94-1.73) | 1.51 (1.06-1.95) | 1.49 (1.05-1.93) | 1.8 (1.27-2.34) | 1.81 (1.27-2.35) |
| *Canterbury* | 2.03 (1.93-2.12) | 1.83 (1.75-1.92) | 1.84 (1.75-1.92) | 1.93 (1.84-2.02) | 1.9 (1.82-1.99) | 1.91 (1.82-2) |
| *South Canterbury* | 2.21 (1.89-2.53) | 1.76 (1.51-2.02) | 1.79 (1.53-2.05) | 1.82 (1.55-2.08) | 1.97 (1.69-2.26) | 1.98 (1.69-2.27) |
| *Southern* | 2.26 (2.14-2.38) | 1.96 (1.85-2.07) | 1.86 (1.76-1.96) | 1.91 (1.8-2.01) | 1.92 (1.81-2.02) | 1.93 (1.82-2.03) |

1. **Elective/Waiting List Procedures:**

|  | ***Crude*** | ***+ Age, Sex*** | ***+ Specialty, Risk*** | ***+ Deprivation*** | ***+ Comorbidity*** | ***+ Ethnicity*** |
| --- | --- | --- | --- | --- | --- | --- |
|  | n/100 (95% CI) | n/100 (95% CI) | n/100 (95% CI) | n/100 (95% CI) | n/100 (95% CI) | n/100 (95% CI) |
| **National Total** | 0.27 (0.26-0.27) | - | - | - | - | - |
|  |  |  |  |  |  |  |
| **District Health Board** | |  |  |  |  |  |
| *Northland* | 0.24 (0.2-0.27) | 0.23 (0.2-0.27) | 0.28 (0.24-0.32) | 0.25 (0.21-0.29) | 0.31 (0.26-0.36) | 0.3 (0.26-0.35) |
| *Waitemata* | 0.17 (0.15-0.19) | 0.18 (0.16-0.2) | 0.2 (0.17-0.22) | 0.21 (0.18-0.24) | 0.21 (0.19-0.24) | 0.21 (0.19-0.24) |
| *Auckland* | 0.35 (0.33-0.37) | 0.38 (0.36-0.4) | 0.31 (0.29-0.33) | 0.31 (0.3-0.33) | 0.25 (0.23-0.26) | 0.25 (0.23-0.26) |
| *Counties-Manukau* | 0.19 (0.17-0.21) | 0.21 (0.19-0.24) | 0.27 (0.24-0.29) | 0.25 (0.23-0.28) | 0.26 (0.23-0.29) | 0.26 (0.23-0.29) |
| *Waikato* | 0.52 (0.48-0.55) | 0.49 (0.46-0.52) | 0.39 (0.37-0.42) | 0.37 (0.35-0.4) | 0.38 (0.36-0.41) | 0.38 (0.35-0.4) |
| *Lakes* | 0.28 (0.23-0.33) | 0.28 (0.23-0.33) | 0.37 (0.3-0.43) | 0.34 (0.28-0.41) | 0.4 (0.33-0.47) | 0.39 (0.32-0.46) |
| *Bay of Plenty* | 0.2 (0.17-0.23) | 0.18 (0.15-0.2) | 0.2 (0.17-0.23) | 0.19 (0.16-0.22) | 0.23 (0.2-0.26) | 0.23 (0.19-0.26) |
| *Tairawhiti* | 0.21 (0.15-0.27) | 0.23 (0.16-0.3) | 0.27 (0.19-0.35) | 0.25 (0.17-0.32) | 0.33 (0.24-0.43) | 0.32 (0.23-0.42) |
| *Hawke's Bay* | 0.24 (0.2-0.27) | 0.21 (0.18-0.23) | 0.28 (0.24-0.32) | 0.27 (0.23-0.3) | 0.3 (0.26-0.34) | 0.3 (0.25-0.34) |
| *Taranaki* | 0.24 (0.2-0.27) | 0.22 (0.18-0.25) | 0.26 (0.22-0.31) | 0.26 (0.22-0.3) | 0.29 (0.25-0.34) | 0.29 (0.24-0.34) |
| *MidCentral* | 0.25 (0.22-0.29) | 0.22 (0.19-0.25) | 0.26 (0.22-0.3) | 0.24 (0.21-0.27) | 0.26 (0.22-0.3) | 0.26 (0.22-0.3) |
| *Whanganui* | 0.17 (0.13-0.21) | 0.15 (0.11-0.19) | 0.2 (0.15-0.25) | 0.19 (0.14-0.23) | 0.25 (0.19-0.31) | 0.25 (0.19-0.31) |
| *Capital and Coast* | 0.33 (0.3-0.35) | 0.33 (0.31-0.36) | 0.27 (0.25-0.29) | 0.27 (0.25-0.29) | 0.26 (0.24-0.28) | 0.26 (0.24-0.28) |
| *Hutt Valley* | 0.14 (0.11-0.16) | 0.16 (0.13-0.2) | 0.21 (0.17-0.25) | 0.21 (0.17-0.25) | 0.23 (0.19-0.28) | 0.23 (0.19-0.28) |
| *Wairarapa* | 0.13 (0.08-0.19) | 0.13 (0.08-0.19) | 0.16 (0.09-0.22) | 0.15 (0.09-0.21) | 0.27 (0.16-0.38) | 0.27 (0.16-0.38) |
| *Nelson/Marlborough* | 0.16 (0.13-0.18) | 0.15 (0.12-0.18) | 0.18 (0.15-0.21) | 0.19 (0.15-0.22) | 0.23 (0.19-0.27) | 0.23 (0.19-0.27) |
| *West Coast* | 0.08 (0.03-0.12) | 0.07 (0.03-0.12) | 0.09 (0.04-0.15) | 0.09 (0.04-0.15) | 0.13 (0.05-0.21) | 0.13 (0.05-0.22) |
| *Canterbury* | 0.21 (0.19-0.23) | 0.21 (0.19-0.22) | 0.21 (0.2-0.23) | 0.22 (0.21-0.24) | 0.23 (0.21-0.25) | 0.23 (0.21-0.25) |
| *South Canterbury* | 0.17 (0.12-0.21) | 0.15 (0.11-0.19) | 0.16 (0.12-0.21) | 0.17 (0.12-0.21) | 0.22 (0.16-0.28) | 0.22 (0.16-0.28) |
| *Southern* | 0.31 (0.28-0.33) | 0.29 (0.26-0.31) | 0.27 (0.24-0.29) | 0.28 (0.25-0.3) | 0.28 (0.25-0.3) | 0.28 (0.25-0.3) |

**Supplementary Material 2:** Māori vs. European 30-day mortality rate ratios (RRs) following **a)** **acute** and **b) elective/waiting list procedures**, by DHB, with iterative adjustment for modelled variables (i.e. tabulation of data presented in Figures 2a and 2b). Data are not presented for those DHBs where fewer than 10 Māori deaths occurred over the study period.

1. **Acute Procedures:**

|  | **Rate Ratio, Māori vs. European (reference group)** | | | | |
| --- | --- | --- | --- | --- | --- |
|  | ***Crude*** | ***+ Age, Sex*** | ***+ Specialty, Risk*** | ***+ Deprivation*** | ***+ Comorbidity*** |
|  | RR (95% CI) | RR (95% CI) | RR (95% CI) | RR (95% CI) | RR (95% CI) |
| **District Health Board** | |  |  |  |  |
| *Northland* | 0.48 (0.39-0.58) | 1.5 (1.22-1.85) | 1.47 (1.2-1.81) | 1.43 (1.15-1.77) | 1.05 (0.85-1.31) |
| *Waitemata* | 0.42 (0.31-0.57) | 1.55 (1.14-2.11) | 1.52 (1.12-2.06) | 1.52 (1.11-2.07) | 1.09 (0.8-1.48) |
| *Auckland* | 1.06 (0.96-1.16) | 1.9 (1.72-2.1) | 1.51 (1.37-1.67) | 1.41 (1.27-1.57) | 1.16 (1.05-1.29) |
| *Counties-Manukau* | 0.51 (0.44-0.6) | 1.76 (1.49-2.08) | 1.64 (1.39-1.94) | 1.44 (1.21-1.72) | 1.09 (0.91-1.3) |
| *Waikato* | 0.73 (0.66-0.8) | 1.7 (1.53-1.89) | 1.56 (1.41-1.74) | 1.51 (1.36-1.68) | 1.13 (1.02-1.27) |
| *Lakes* | 0.45 (0.35-0.58) | 1.47 (1.13-1.91) | 1.44 (1.11-1.87) | 1.34 (1.02-1.75) | 0.97 (0.74-1.27) |
| *Bay of Plenty* | 0.32 (0.26-0.41) | 1.35 (1.05-1.74) | 1.34 (1.04-1.72) | 1.34 (1.03-1.73) | 1.12 (0.86-1.44) |
| *Tairawhiti* | 0.34 (0.24-0.48) | 1.04 (0.72-1.5) | 1.04 (0.72-1.51) | 0.93 (0.64-1.37) | 0.8 (0.54-1.18) |
| *Hawke's Bay* | 0.5 (0.4-0.61) | 1.93 (1.55-2.41) | 1.91 (1.53-2.39) | 1.83 (1.46-2.31) | 1.44 (1.14-1.82) |
| *Taranaki* | 0.39 (0.27-0.56) | 1.3 (0.89-1.89) | 1.27 (0.87-1.85) | 1.23 (0.84-1.79) | 0.95 (0.65-1.39) |
| *MidCentral* | 0.4 (0.29-0.54) | 1.36 (0.99-1.88) | 1.33 (0.96-1.84) | 1.28 (0.92-1.77) | 0.98 (0.71-1.36) |
| *Whanganui* | 0.39 (0.27-0.59) | 1.74 (1.15-2.64) | 1.81 (1.19-2.75) | 1.77 (1.16-2.7) | 1.28 (0.84-1.96) |
| *Capital and Coast* | 0.71 (0.61-0.83) | 1.39 (1.18-1.63) | 1.21 (1.03-1.42) | 1.15 (0.97-1.36) | 0.94 (0.79-1.11) |
| *Hutt Valley* | 0.23 (0.15-0.35) | 1.05 (0.67-1.65) | 1.11 (0.7-1.74) | 1.27 (0.79-2.03) | 0.77 (0.49-1.22) |
| *Wairarapa* | Not Calculated | | | | |
| *Nelson/Marlborough* | 0.46 (0.28-0.75) | 1.93 (1.17-3.2) | 1.74 (1.05-2.87) | 1.68 (1.02-2.79) | 1.49 (0.9-2.46) |
| *West Coast* | Not Calculated | | | | |
| *Canterbury* | 0.74 (0.62-0.9) | 1.98 (1.63-2.41) | 1.87 (1.54-2.27) | 1.77 (1.45-2.15) | 1.46 (1.21-1.78) |
| *South Canterbury* | Not Calculated | | | | |
| *Southern* | 0.26 (0.18-0.37) | 0.71 (0.5-1.02) | 0.7 (0.49-1) | 0.65 (0.45-0.94) | 0.62 (0.43-0.89) |

1. **Elective/Waiting List Procedures:**

|  | **Rate Ratio, Māori vs. European (reference group)** | | | | |
| --- | --- | --- | --- | --- | --- |
|  | ***Crude*** | ***+ Age, Sex*** | ***+ Specialty, Risk*** | ***+ Deprivation*** | ***+ Comorbidity*** |
|  | RR (95% CI) | RR (95% CI) | RR (95% CI) | RR (95% CI) | RR (95% CI) |
| **District Health Board** | |  |  |  |  |
| *Northland* | 0.85 (0.61-1.18) | 2.41 (1.71-3.39) | 2.53 (1.79-3.57) | 2.35 (1.64-3.35) | 1.58 (1.1-2.28) |
| *Waitemata* | 0.53 (0.31-0.89) | 1.5 (0.87-2.56) | 1.49 (0.87-2.56) | 1.43 (0.83-2.47) | 1.2 (0.67-2.15) |
| *Auckland* | 1.13 (0.97-1.32) | 2.05 (1.75-2.41) | 1.79 (1.53-2.11) | 1.61 (1.36-1.9) | 1.24 (1.04-1.46) |
| *Counties-Manukau* | 1.17 (0.9-1.51) | 2.78 (2.12-3.65) | 2.73 (2.08-3.6) | 2.66 (1.99-3.55) | 1.81 (1.35-2.43) |
| *Waikato* | 0.82 (0.7-0.97) | 1.69 (1.43-2) | 1.55 (1.31-1.84) | 1.5 (1.26-1.79) | 1.01 (0.85-1.21) |
| *Lakes* | 0.57 (0.38-0.86) | 1.49 (0.97-2.28) | 1.73 (1.13-2.67) | 1.88 (1.2-2.93) | 1.33 (0.85-2.1) |
| *Bay of Plenty* | 0.57 (0.38-0.84) | 2.07 (1.36-3.14) | 2.11 (1.39-3.2) | 1.87 (1.22-2.87) | 1.5 (0.98-2.3) |
| *Tairawhiti* | 0.54 (0.29-1.01) | 1.56 (0.8-3.03) | 1.75 (0.89-3.45) | 1.83 (0.91-3.67) | 1.55 (0.77-3.15) |
| *Hawke's Bay* | 0.73 (0.52-1.05) | 1.87 (1.3-2.71) | 2 (1.38-2.9) | 2.07 (1.41-3.03) | 1.53 (1.03-2.27) |
| *Taranaki* | 0.58 (0.33-1) | 1.48 (0.84-2.61) | 1.63 (0.92-2.88) | 1.46 (0.82-2.6) | 0.96 (0.54-1.71) |
| *MidCentral* | 0.93 (0.62-1.41) | 2.18 (1.43-3.34) | 2.22 (1.45-3.41) | 2.06 (1.34-3.16) | 1.14 (0.73-1.77) |
| *Whanganui* | 1.04 (0.58-1.85) | 2.18 (1.18-4) | 3.13 (1.68-5.82) | 2.44 (1.3-4.6) | 1.98 (0.97-4.04) |
| *Capital and Coast* | 1.02 (0.81-1.29) | 1.97 (1.55-2.51) | 1.87 (1.47-2.38) | 1.75 (1.36-2.24) | 1.35 (1.05-1.73) |
| *Hutt Valley* | 0.52 (0.27-0.99) | 1.74 (0.87-3.47) | 1.92 (0.98-3.77) | 1.52 (0.76-3.04) | 1.36 (0.68-2.7) |
| *Wairarapa* | Not Calculated | | | | |
| *Nelson/Marlborough* | Not Calculated | | | | |
| *West Coast* | Not Calculated | | | | |
| *Canterbury* | 0.74 (0.54-1.02) | 1.8 (1.3-2.5) | 1.71 (1.24-2.37) | 1.67 (1.21-2.32) | 1.37 (0.99-1.9) |
| *South Canterbury* | Not Calculated | | | | |
| *Southern* | 0.45 (0.28-0.71) | 1.08 (0.68-1.73) | 1.12 (0.7-1.78) | 1.03 (0.65-1.65) | 0.92 (0.57-1.46) |
